# Prevalence of Amiodarone-Induced Thyrotoxicosis and Associated Risk Factors in Japanese Patients

**DOI:** 10.1155/2014/534904

**Published:** 2014-06-25

**Authors:** Toyoyoshi Uchida, Takatoshi Kasai, Atsutoshi Takagi, Gaku Sekita, Koji Komiya, Kageumi Takeno, Nayumi Shigihara, Kazunori Shimada, Katsumi Miyauchi, Yoshio Fujitani, Hiroyuki Daida, Hirotaka Watada

**Affiliations:** ^1^Department of Metabolism & Endocrinology, Juntendo University Graduate School of Medicine, 2-1-1 Hongo, Bunkyo-ku, Tokyo 113-8421, Japan; ^2^Department of Cardiology, Juntendo University Graduate School of Medicine, Bunkyo-ku, Tokyo 113-8421, Japan

## Abstract

Amiodarone is a widely used agent for life-threatening arrhythmias. Although amiodarone-induced thyrotoxicosis (AIT) is a major adverse effect that can cause recurrence of arrhythmias and exacerbation of heart failure, risk factors for AIT among amiodarone-treated Japanese patients have not been elucidated. Here, we investigated the prevalence and predictive factors for AIT. The study subjects were 225 patients treated with amiodarone between 2008 and 2012, who were euthyroid before amiodarone therapy. All patients with AIT were diagnosed by measurement of thyroid hormones and ultrasonography. Among the 225 subjects, 13 patients (5.8%) developed AIT and all the patients were classified as Type 2 AIT. Baseline features of patients with AIT were not different from those who did not develop AIT, except for age (AIT, 55.1 ± 13.8, non-AIT, 68.1 ± 12.0 years, *P* < 0.001). Multivariate analyses using the Cox proportional hazard model identified age as the sole determinant of AIT (hazard ratio: 0.927, 95% confidence interval: 0.891–0.964). Receiver operating characteristic curve analysis identified age of 63.5 years as the cutoff value for AIT with sensitivity of 70.3% and specificity of 69.2%. In summary, this study showed that the prevalence of AIT is 5.8% in Japanese patients treated with amiodarone and that young age is a risk factor for AIT.

## 1. Introduction

Amiodarone is superior to other drugs in maintaining sinus rhythm in patients with persistent or paroxysmal atrial fibrillation and in suppressing life-threatening arrhythmias in patients with left ventricular dysfunction and postmyocardial infarction [1, 2]. However, at clinical doses, amiodarone has several side effects such as interstitial pneumonia, severe hepatic dysfunction, and corneal disorders. Thyroid dysfunction caused by rich iodine contained in amiodarone is another major side effect. While the prevalence of thyroid dysfunction is reported to be high among the amiodarone-treated patients [3–5], amiodarone-induced thyrotoxicosis (AIT) is a serious side effect as it can cause recurrence of arrhythmias and heart failure that often require hospitalization [6, 7].

The reported prevalence of AIT varies widely, from 0.8–2.2% [8, 9] to 4.0–13.6% [10–15], to as high as 20.1–37.8% [16–18]. This variation is partly due to the definition of AIT used in these studies. Indeed, AIT was defined as new-onset of symptomatic thyrotoxicosis during amiodarone administration in the studies showing low prevalence of AIT [8, 9]. On the other hand, it was defined as suppression of TSH level and elevation of free triiodothyronine (FT3) and/or free thyroxine (FT4) level during amiodarone treatment in studies showing intermediate prevalence of AIT [10–15] and high levels of triiodothyronine (T3) and/or thyroxine (T4) during amiodarone treatment in studies showing high prevalence of AIT [16, 17]. These facts suggest that the reported high prevalence rate of AIT could be explained by the inclusion of patients with modest basal thyroid functional abnormality and no substantial thyroid dysfunction. Previously, Martino et al. [19] reported higher prevalence of AIT in iodine-poor areas compared to that in iodine-rich areas. However, according to various kinds of reports, the prevalence of AIT in iodine-poor areas was 9.6–20.5% [10, 18, 19] and that in iodine-rich areas was 2.4–37.5% [11–17, 19]. These evidences suggested that iodine intake has no significant effect on the prevalence of AIT. This difference could be mainly due to the different definition of AIT. At least, proper estimation of the prevalence of AIT should be based on the examination of patients free from obvious preexisting thyroid disorders before starting amiodarone therapy. However, in many previous studies, the thyroid status was not investigated before treatment with amiodarone [8, 9, 11, 12, 17, 19].

AIT is divided into two subtypes, types 1 and 2 AIT. Ultrasound findings are helpful in the diagnosis of subtypes of AIT [3, 4]. Type 1 AIT shows increased thyroid hormone synthesis from an autonomously functioning thyroid. The ultrasonography findings in type 1 AIT often include multinodular goiter and enhancement of color flow Doppler sonography (CFDS). On the other hand, type 2 AIT shows almost normal ultrasound findings despite increased thyroid hormone release by thyroid destructive process. However, in most previous studies, correct evaluation of thyrotoxicosis to diagnose AIT and the exclusion of other thyroid abnormalities did not seem to be systematically performed [8–12, 16, 17, 19].

In the present study, we retrospectively estimated the prevalence of AIT in a cohort group of patients who were systematically diagnosed with AIT. Then, we searched for the predictors for the onset of AIT among Japanese patients who were being treated with amiodarone.

## 2. Subjects and Methods

### 2.1. Patients

**Figure 1:**
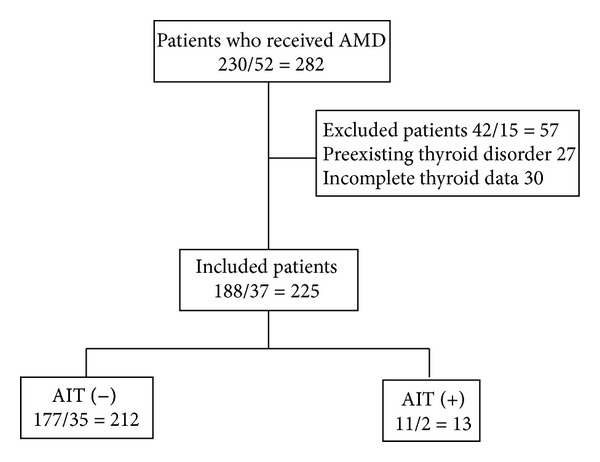
Retrospective cohort study design. Subgroups show the* group name* and registered number of* male/female = total patients*.

Between October 1, 2008 and July 31, 2012, 286 consecutive Japanese patients (males 230, females 52) were being treated with oral amiodarone and followed at Juntendo University Hospital. Among them, we excluded 57 patients (males 42, females 15) due to obvious preexisting thyroid disorders (*n* = 27) or for not undergoing laboratory assessment of thyroid function before starting amiodarone (*n* = 30). The remaining 225 patients (males 188, females 37) were enrolled in this study (Figure 1). We retrospectively collected the data of serial thyroid hormone levels, treatment period, and cumulative dosage of amiodarone until July 31, 2012. The ethics committee of Juntendo University approved the study protocol.

### 2.2. Definition of Underlying Cardiac Disorders

Chronic heart failure was diagnosed based on the clinical examination by cardiologists and defined as heart failure due to cardiomyopathy or valvular heart diseases with New York Heart Association class ≥II for ≥6 months. Left ventricular systolic dysfunction was defined as left ventricular ejection fraction of ≤35%. Ischemic heart disease was defined as history of angina pectoris and myocardial infarction.

### 2.3. Measurement of Serological Makers

Blood samples were collected from all study subjects. Serum FT4, FT3, and TSH values were measured using commercial electrochemiluminescence immunoassay kits (Roche Diagnostics, Tokyo, Japan; FT4 normal range, 1.00–1.70 ng/dL; FT3 normal range, 2.40–4.50 pg/mL; TSH normal range 0.56–4.30 *μ*IU/mL). Serum thyrotropin receptor antibody (TRAb) was measured using a two-step radioreceptor assay (DYNO test TRAb; Yamasa Corp, Tokyo: normal range <1.0 IU/L).

### 2.4. Diagnostic Criteria of AIT

AIT was diagnosed when the patients fulfilled all the following criteria [3]; (1) treated with amiodarone at the time of the study. (2) Reproducible thyrotoxicosis at least 2 week interval with/without new symptoms such as body weight loss, palpitation, and dyspnea. (3) Exclusion of other drugs that could induce thyrotoxicosis. Mainly based on ultrasonography findings, patients with AIT were divided into two subtypes: Type 1 AIT with hyperfunctioning and hypervascular thyroid and/or nodular goiter and Type 2 AIT with destructive thyroid but no hypervascularity. Thyroid autoantibodies and TRAb were also used for the diagnosis of subtype of AIT.

### 2.5. Statistical Analysis

Results are presented as mean and standard deviation (SD) or median with interquartile range (IQR) and compared using Student's *t*-test or Mann-Whitney *U*-test, as appropriate. Categorical data were displayed as frequencies and compared using the Chi-square test or Fisher's exact test. To determine factors that can predict the onset of AIT among the baseline variables, Cox proportional hazards models were used. First, univariate analyses were conducted using baseline independent variables: gender, age, TSH, FT3, FT4, chronic heart failure, left ventricular systolic dysfunction, ischemic heart disease, nonsustained ventricular tachycardia (NSVT), ventricular fibrillation, arterial fibrillation, and use of *α*-blockers, *β*-blockers, antiangiotensin agents (angiotensin receptor blockers, angiotensin converting enzyme inhibitors), antialdosterone agents (spironolactone, eplerenone), diuretics, antithrombotic agents, nitrates, calcium channel blockers, and other antiarrhythmic agents. In addition, the cumulative dose of amiodarone during the follow-up period was also included as an independent variable although it is not a baseline variable. Variables that showed *P* < 0.10 on univariate analyses were entered into a multivariate Cox proportional hazards regression analysis. The assumption of proportional hazards was assessed by visual judgment of the log-minus-log survival plots. The best cutoff values for continuous variables predicting risk of AIT were generated with receiver operating characteristics (ROC) curves. *P* values <0.05 denoted the presence of significant difference. All statistical analyses were computed using The Statistical Package for Social Sciences (SPSS Inc., Chicago, IL).

## 3. Results

### 3.1. Prevalence and Clinical Features of Patients Treated with Amiodarone

Among the 225 enrolled patients in this study, 13 patients (males 11, females 2) were diagnosed with amiodarone-induced AIT (5.8%) during a median follow-up period of 1295 days. All patients were diagnosed as Type 2 AIT. Regarding other side effects, 67 patients developed amiodarone-induced hypothyroidism and a total of 9 patients developed other side effects as follows: 3 patients with moderate hepatic dysfunction, 2 patients with mild interstitial pneumonia, 2 patients with corneal disorder, and 2 patients with moderate renal dysfunction in the enrolled 225 patients.

### 3.2. Predictors of Onset of AIT

In comparison with baseline data, patients with AIT were younger than patients without AIT (AIT; 55.1 ± 13.8, non AIT; 68.1 ± 12.0 years (yrs), resp., *P* < 0.001, Table 1). There were no statistical differences in gender, observation period, cumulative amiodarone dosage, baseline thyroid function, and background cardiac disorders and arrhythmias between the two groups. Univariate Cox proportional hazards regression analyses showed that amiodarone-induced AIT correlated only with patient's age (Table 2). Stepwise multivariate Cox's proportional hazards regression analysis also identified patient's age as the sole significant risk factor for AIT (*P* < 0.0001, hazard ratio (HR) 0.927 (95% confidence interval (CI): 0.891–0.964)). ROC curve analysis of patient's age using data of patients with/without the onset of AIT showed that 63.5 years was the cutoff value (sensitivity: 70.3%, specificity: 69.2%). We also conducted another univariate Cox proportional hazards regression analysis using categorized age data (<63.5 or ≥63.5 yrs) as risk factor of Type2 AIT. The results of this analysis showed that age <63.5 years at the start of amiodarone therapy was a predictor for the onset of AIT (*P* = 0.023, HR 3.922, 95% CI: 1.204–12.775).

## 4. Discussion

In this study, we investigated the prevalence of AIT in Japanese patients treated with amiodarone who were confirmed to be euthyroid before the commencement of amiodarone therapy and the risk factors for the onset of AIT. The prevalence of AIT was 5.8% among our study patients and all AIT patients were diagnosed as Type2 AIT. Patients with AIT were younger than those without AIT. The results of multivariate Cox's proportional hazards regression analysis identified age as the only sole determinant of AIT. Our results suggest that AIT is not rare in Japanese patients treated with amiodarone, especially in patients aged less than 63.5 years.

Previous studies reported wide range of prevalence of AIT (1 to 38%), which is probably due to various definitions of AIT [8–19]. Among these studies, Ahmed et al. [13] conducted a study using definition of AIT similar to that applied in the present study. They excluded preexisting thyroid disease before starting amiodarone, confirmed reproducible thyrotoxicosis (suppression of TSH level and elevation of FT3 and/or FT4 level), and used ultrasound for the diagnosis of thyrotoxicosis. Their study showed AIT prevalence of 7.5% among 303 Dutch patients treated with amiodarone [13]. In addition, two recent studies in Asia calculated the prevalence of AIT. In both studies, preexisting thyroid disease before starting amiodarone was excluded and reproducible thyrotoxicosis was confirmed. The study whose subjects are 390 Hong-Kong Chinese patients treated by amiodarone showed that the prevalence of AIT is 6.0% and the study of 527 Taiwan patients showed its prevalence to be 4.0% [14, 15]. According to the WHO global database on iodine deficiency [20] and the Dietary Reference Intakes for Japanese 2010 [21], iodine status of all these countries was evaluated as adequate. Accordingly, the studies using proper diagnosis process of AIT in the countries under similar status of iodine intake showed the similar prevalence of AIT to this study (5.8%).

Sato et al. [22, 23] were the first Japanese group to describe the characteristics of AIT and to publish case reports of recurrent AIT among Japanese patients treated with amiodarone. The former study was done by well evaluated pre-/postthyroid status including ultrasonography findings, but they reported the number of cases without showing the total numbers of patients treated with amiodarone in these studies; thus, the prevalence of AIT among the patients treated by amiodarone was not able to be calculated. Shiga et al. [12] were the first group that analyzed the prevalence of AIT among Japanese patients, and reported a prevalence of 12.5%. However, they did not report thyroid status before treatment with amiodarone nor excluded thyroid abnormalities. Accordingly, our study is the first to show the prevalence of AIT in Japanese patients calculated by proper diagnostic process.

Our results showed that patients with AIT were significantly younger than patients without AIT, and the risk factor for the onset of AIT was younger age (<63.5 years, HR = 3.9). Although previous studies did not use risk factor analysis [4, 10, 11], others reported that patients with AIT were significantly younger than those without AIT [7, 13, 16]. We speculate that the difference in the risk factors of AIT among the studies is mainly due to study design. Indeed, Ahmed et al. identified young age (62 years) as the only risk factor of AIT using similar definition of AIT to our study [13]. Additionally, the other two studies in Asia also indicated younger age as the only risk factor of AIT [14, 15]. Taken together, using systematic diagnosis criteria of AIT, the risk factors of AIT seem to be identical to in the other recent study.

It is difficult to speculate on how young age enhances the susceptibility to AIT. To our knowledge, there are no previous epidemiological or clinical data on this issue. Further studies are needed to determine the mechanism by which amiodarone treatment increases the likelihood of development of thyrotoxicosis in young patients with cardiac disorders, but not elderly people.

The present study has several limitations. First, the number of the patients was small. Second, the study was a single center observational study of daily clinical practice. Third, the iodine status of the population is presumably the main reason why all of the observed cases are type 2, but we did not estimate the iodine status of this population. Fourth, we did not measure autoimmune thyroid antibodies before the start of amiodarone therapy, thus, we could not evaluate these antibodies as other risk factors. Further studies are needed to confirm our findings and identify other risk factors.

In conclusion, the prevalence of AIT and risk factors for AIT in Japanese patients treated with amiodarone are similar to previous reports using proper diagnostic process of AIT. The results call for careful monitoring of thyroid function especially in amiodarone-treated young patients.

## Figures and Tables

**Table 1:** Clinical features of patients treated with amiodarone with/without the onset of AIT.

	AIT (−)	AIT (+)	*P*
Patients number	212	13	
Gender (Male/Female)	177/35	11/2	0.915
Age (yrs)	68.1 ± 12.0	55.1 ± 13.8	<0.001
TSH (*μ*U/mL)	2.00 ± 1.27	1.66 ± 0.64	0.337
Free T3 (pg/mL)	2.52 ± 0.58	2.65 ± 0.42	0.428
Free T4 (ng/dL)	1.32 ± 0.21	1.36 ± 0.24	0.484
Observation period (days)	1333 (709.5–2464)	970 (899–1462)	0.362
Cumulative dosage of AMD (g)	137 (52–265)	194 (142–252)	0.256
Cardiac disorders			
Chronic heart failure	208	12	0.930
Left ventricular systolic dysfunction	126	6	
Ischemic heart disease	96	5	
Arrhythmia			
NSVT	162	11	0.410
Ventricular fibrillation	18	2	
Atrial fibrillation	44	1	
Cardiac medications			
*α*-blockers	2	0	0.725
*β*-blockers	170	12	0.28
Anti-angiotensin agents	143	10	0.477
Anti-aldosteron agents	84	8	0.119
Diuretics	131	8	0.985
Antithrombotic agents	179	10	0.473
Nitrates	32	2	0.977
Calcium channel blockers	35	2	0.915
Other antiarrhythmic agents	14	0	0.339

NSVT: nonsustained ventricular tachycardia.

Data of observation period and cumulative dosage of amiodarone (AMD) are expressed as median (IQR). Other data are expressed as mean ± SD.

**Table 2:** Results of univariate Cox proportional hazards regression analysis.

	*P*	HR (95% CI^¶^)
Gender	0.965	0.967 (0.214–4.366)
Age	<0.0001	0.927 (0.891–0.964)
TSH	0.457	0.814 (0.472–1.402)
Free T3	0.476	1.388 (0.563–3.422)
Free T4	0.925	1.137 (0.077–16.802)
Cumulative dosage of AMD	0.201	1.000 (0.999–1.000)
Cardiac disorders		
Chronic heart failure	0.732	0.048 (0.000–1651676)
Low left ventricular function	0.277	1.834 (0.615–5.463)
Ischemic heart disease	0.859	1.107 (0.361–3.389)
Arrhythmia		
NSVT	0.607	0.671 (0.147–3.071)
Ventricular fibrillation	0.218	0.298 (0.095–1.360)
Atrial fibrillation	0.928	1.099 (0.140–8.606)
Cardiac medications		
*α*-blocker	n/a	n/a
*β*-blocker	0.214	0.274 (0.035–2.115)
Anti-angiotensin agents	0.534	0.664 (0.183–2.413)
Anti-aldosteron agents	0.246	0.516 (0.169–1.578)
Diuretics	0.889	1.083 (0.354–3.3118)
Antithrombotic agents	0.466	1.616 (0.444–5.882)
Vasodilators	0.888	1.114 (0.247–5.028)
Calcium channel blocker	0.914	1.087 (0.241–4.904)
Antiarrhythmic agents	0.478	22.97 (0.003–132741)

NSVT: nonsustained ventricular tachycardia.

^¶^Confidence interval.

n/a: no assign.

## Abbreviations

AIT: Amiodarone-induced thyrotoxicosis
(F)T3: (free) triiodothyronine
(F)T4: (free) thyroxine
TSH: Thyrotropin.
